# Editorial Note: Predicting corporate credit risk: Network contagion via trade credit

**DOI:** 10.1371/journal.pone.0347629

**Published:** 2026-04-21

**Authors:** The PLOS One Editors

## Notice of Republication

This article [1] was republished on April 3, 2026, to address an issue involving the published peer review history. The article content has not been changed.
